# Protein expression and gene polymorphism of CXCL10 in patients with colorectal cancer

**DOI:** 10.3892/br.2014.255

**Published:** 2014-03-17

**Authors:** JAN DIMBERG, MARITA SKARSTEDT, STURE LÖFGREN, NIKLAS ZAR, ANDREAS MATUSSEK

**Affiliations:** 1 Department of Natural Science and Biomedicine, University College of Health Sciences, Jönköping, Småland SE-55111, Ryhov County Hospital, Jönköping, Småland SE-55185, Sweden; 2 Department of Clinical Microbiology, Ryhov County Hospital, Jönköping, Småland SE-55185, Sweden; 3 Department of Surgery, Ryhov County Hospital, Jönköping, Småland SE-55185, Sweden; 4 Department of Laboratory Services, Ryhov County Hospital, Jönköping, Småland SE-55185, Sweden

**Keywords:** CXCL10, gene polymorphism, protein expression, colorectal cancer

## Abstract

Chemokines (chemotactic cytokines) promote leukocyte attraction to sites of inflammation and cancer. Certain chemokines promote and regulate neoplastic progression, including metastasis and angiogenesis. One such chemokine, CXCL10, was found to be expressed in colorectal cancer (CRC) tissue. To gain insight into the prognostic significance of CXCL10, we investigated whether the levels of this chemokine were altered in the colorectal tissue or plasma of CRC patients. Using Luminex technology for protein analyses, we observed a significantly higher CXCL10 protein level in cancer tissue compared to that in paired normal tissue. Moreover, significantly higher plasma levels of CXCL10 were detected in patients compared to those in control subjects and the plasma levels of CXCL10 in disseminated disease were found to be significantly higher compared to those in localized disease. The single-nucleotide polymorphism rs8878, which has been described in exon 4 in the 3′-untranslated region of the CXCL10 gene, was investigated using a TaqMan system. There were significant differences in genotype distribution and allelic frequencies between CRC patients and control subjects. In conclusion, altered CXCL10 protein concentrations in CRC tissues or plasma and the rs8878 genotype variant of CXCL10 may contribute to the prediction of clinical outcome.

## Introduction

Chemokines (chemotactic cytokines) promote leukocyte attraction to sites of inflammation and cancer (1–3). Certain chemokines promote and regulate neoplastic progression, including metastasis and angiogenesis (4,5).

Chemokine CXCL10, also referred to as interferon (IFN)-γ inducible protein-10, is a protein expressed and secreted by various types of cells, such as monocytes/macrophages, endothelial cells, fibroblasts and dendritic cells (6). CXCL10 acts via the CXCR3 receptor and is a potent chemoattractant for various immune cells, including activated T cells, such as T helper (Th1) cells, macrophages and natural killer cells (4,7–9). Moreover, CXCL10 suppresses neovascularization, acts as an angiostatic chemokine and induces apoptosis and cell growth inhibition (4,10).

CXCL10 is constitutively expressed in normal human colonic epithelium (7,11) and was shown to be elevated as well as suppressed in colorectal cancer (CRC) compared to adjacent normal tissue (12,13). Moreover, high serum levels of CXCL10 have been associated with poor prognosis in CRC (14).

A single-nucleotide polymorphism (SNP), rs8878 (T>C), has been described in exon 4 in the 3′-untranslated region of the CXCL10 gene (15) and may affect the stability of the related mRNA. This SNP was previously evaluated in patients with Alzheimer’s disease (AD) (15) and multiple sclerosis (MS) (16) to determine whether CXCL10 is a susceptibility gene for AD and MS. However, according to those results, such an association was not verified.

Local immunoregulation mediated by infiltration of inflammatory cells into colorectal adenocarcinomas is considered crucial for tumour progression (1–3). CXCL10 may play a partial antitumourigenic role, possibly through driving the Th1 response. The role of the matrix metalloproteinases (MMPs) in the progression of malignancies has been well documented (17,18). CXCL10 was shown to promote the secretion or activity of MMPs in CRC cells and monocytes, thus enhancing the malignancy of the tumour microenvironment (19,20).

To the best of our knowledge, available data on the expression profile of CXCL10 in human CRC tissue and plasma are limited. Therefore, we determined the protein expression of this chemokine in CRC patients and its association with clinical parameters. We also screened for CXCL10 gene polymorphism (rs8878) to evaluate a possible association with the clinical outcome of CRC.

## Materials and methods

### Patients and controls

Blood samples from 366 consecutive CRC patients from Southeastern Sweden were collected during surgical resection for primary colorectal adenocarcinoma at the Department of Surgery, Ryhov County Hospital, Jönköping, Sweden. The clinicopathological characteristics of the patients were retreived from the surgical and pathological records. The patient group included 199 men and 167 women, with a mean age of 70 years (range, 29–93 years). The tumours were localized in the colon (n=204) or rectum (n=162) and were classified according to the Dukes’ classification system as stage A (n=63), B (n=138), C (n=108) and D (n=57). Blood donors (n=545) from Ryhov County Hospital, from the same geographical region as the CRC patients and without known CRC history, were selected as controls. The control group included 270 men and 275 women, with a mean age of 64 years (range, 30–80 years). All the blood samples were centrifuged to separate the plasma and the blood cells and stored at −70°C.

The study protocol was approved by the local Ethics Committee and informed consent was obtained from all the participants.

### Tissue samples and lysates

This study utilized tumour and matched normal tissue samples available from 99 of the CRC patients, of whom 50 were men and 49 women, with a mean age of 68 years (range, 29–90 years). The tumours were classified as Dukes’ stage A (n=18), B (n=33), C (n=23) and D (n=25). The tumours were localized in the colon (n=54) or rectum (n=45). Tumour tissue and adjacent normal mucosa (~5 cm from the tumour) from each patient were excised and immediately frozen at −70°C until analysis.

Tumour tissue, paired normal mucosa and cell lines were homogenised in ice-cold RIPA lysis buffer (Santa Cruz Biotechnology, Inc., Santa Cruz, CA, USA), containing a protease inhibitor cocktail, according to the manufacturer’s instructions. The lysate was placed on ice for 30 min and then centrifuged at 18,000 × g for 10 min. The protein content of the supernatant fluid was determined for each sample using the Bradford protein assay (Bio-Rad Laboratories, Hercules, CA, USA).

### Plasma samples

Of the CRC patients and controls, 104 and 92, respectively, were available for chemokine analysis. The CRC patient group comprised 52 men and 52 women, with a mean age 68 years (range, 29–90 years). The patients were classified according to the Dukes’ classification system as stage A (n=18), B (n=34), C (n=26) and D (n=26). A total of 46 tumours were located in the rectum and 58 in the colon. The control samples included plasma from 48 men and 44 women, with a mean age of 63 years (range, 56–68 years).

### Quantification of CXCL10 protein in tissue and plasma

CXCL10 was measured in tissue and plasma using Luminex technology (Bio-Rad, Hercules, CA, USA). The tissue level of CXCL10 was expressed as pg/mg of protein. The plasma CXCL10 levels of CRC patients and controls were expressed as pg/ml.

### DNA extraction and genotype determination

DNA was isolated from the blood of all the patients and control subjects using the QIAamp DNA Blood kit (Qiagen, Hilden, Germany). The DNA samples were genotyped using the 5′-exonuclease allelic discrimination assay (Applied Biosystems, Foster City, CA, USA). The TaqMan SNP genotyping assay (Applied Biosystems) was used for the analysis of the rs8878 CXCL10 genotype. DNA (10 ng) was amplified using the 7500 Fast Real-Time PCR system (Applied Biosystems) in a total volume of 12 μl containing TaqMan Universal PCR Master mix (Applied Biosystems), included in the SNP genotyping assay. Amplification was performed using an initial cycle at 50°C for 2 min, followed by 1 cycle at 95°C for 10 min and finally 40 cycles at 95°C for 15 sec and at 60°C for 1 min. A post-PCR endpoint reading was performed on each plate. The manual calling option in the allelic discrimination application ABI PRISM 7500 SDS software, version 1.3.1 (Applied Biosystems), was then used to assign genotypes.

### Statistical analysis

The differences in the frequencies of the CXCL10 gene polymorphism between CRC patients and control subjects and between clinical data within the CRC subgroup were analyzed using the Chi-square test. The Hardy-Weinberg equilibrium was assessed for the genotypes. The comparisons between groups regarding tissue and plasma levels of CXCL10 were performed with non-parametric tests. The Wilcoxon’s signed-rank test and the Mann-Whitney U test were used for the analysis of the related and independent parameters. The correlations between parameters were analyzed according to the Spearman’s rank correlation test. Statistical analyses were performed using SPSS software for Windows, version 14.0 (SPSS, Inc., Chicago, IL, USA). P<0.05 was considered to indicate a statistically significant difference.

## Results

### Protein levels of CXCL10 in colorectal tissue

The levels of CXCL10 in tumour tissue were significantly (P<0.001) higher compared to those in normal paired tissue (Table I). The assessment of the relative expression (tumour vs. normal paired tissue) revealed that CXCL10 was upregulated in 86% (85/99) of the cases. Furthermore, we observed that the protein levels of CXCL10 in tumour and normal tissue were significantly positively correlated (r=0,60, P<0.001) (data not shown).

There was no association with clinical characteristics, such as age, gender, Dukes’ stage and tumour location.

### Plasma levels of CXCL10

The levels of plasma CXCL10 from 104 patients were significantly (P<0.01) higher compared to those in control subjects (Table I); however, the plasma levels were not correlated to the levels in cancer tissue (data not shown).

As regards disease stage, the CRC patients were divided into two subgroups: Dukes’ stage A+B (localized disease) and C+D (disseminated disease). The plasma levels of CXCL10 in patients with Dukes’ C+D disease (median, 946 pg/ml; range, 108–44,128) were significantly (P=0.031) higher compared to those in patients with Dukes’ A+B disease (median, 697 pg/ml; range, 46–6,940).

There was no association with other clinical characteristics, such as age, gender and tumour location (data not shown).

### CXCL10 genotype

There were significant differences (P=0.037) in the genotype distribution and allelic frequencies of the CXCL10 gene polymorphism between the CRC patients and the control subjects (Table II). When assessing these differences, the rate of allele T was found to be 52.6% in the CRC patients compared to 47.6% in the control subjects, with an odds ratio of 1.22 (95% confidence interval: 1.01–1.47). The genotype and allelic distributions in CRC patients and controls were not found to be associated with age, gender and plasma levels of CXCL10 or tumour location, stage and tumour tissue levels of CXCL10. There was no significant deviation from the Hardy-Weinberg equilibrium in the patient or the control group.

## Discussion

The chemokine CXCL10 has been implicated in the development of CRC. Previous studies reported tumour-promoting as well as antimalignant functions of CXCL10 (5,12–14,19). However, the expression profile of CXCL10 in CRC patients has not been fully evaluated. In this study, we demonstrated that lysates from cancer tissue exhibited significantly higher levels of CXCL10 protein compared to those in paired normal tissues. This finding is consistent with data from a previous study using an immunohistochemical approach (14). In our study, the CXCL10 levels in tissue from CRC patients were not correlated with Dukes’ stage. These results are in agreement with data reported by another study, based on immunohistochemistry, which was limited to disease stage II and III (13), according to the International Union Against Cancer (UICC) classification system. CRC is a heterogeneous disease, with different molecular pathways leading to different phenotypes (21). The mechanism through which CXCL10 activity is affected by disease stage in this context has not been fully elucidated. CXCL10 may be expressed and secreted by various types of cells, such as monocytes/macrophages, endothelial cells, fibroblasts and dendritic cells (6), thereby masking the local changes in CXCL10 levels attributed to cancer cells. Moreover, the cytokines interleukin-1β and IFN-γ are upregulated in CRC tissues and were shown to induce the production of CXCL10 (6,12,22), which is an event that may be controlled by a local inflammatory process, without any association to tumour stage. Of note, we observed that the protein levels of CXCL10 in cancer and normal tissues were significantly positively correlated, which may reflect individual differences in the basal expression of CXCL10.

The plasma levels of CXCL10 in CRC patients were significantly higher compared to those in controls and were significantly higher in patients with disseminated disease compared to those in patients with localized disease. This finding is in agreement with a previous study reporting that the circulating levels of CXCL10 were significantly increased with progression of disease stage according to the UICC classification (14). However, our findings indicate that plasma CXCL10 levels do not reflect the local level of CXCL10 in cancer tissue but are correlated well with disease stage. A likely explanation for this may be that the leakage of CXCL10 from cancer tissue is controlled by increased MMP activity due to the higher disease stage.

In the present study, we observed that the genotype and allelic distributions of the SNP rs8878 (T>C) in CRC patients and control subjects were statistically different. However, we were unable to detect any association between genotype and plasma or tissue concentration of CXCL10. The mechanism of action of the investigated SNP remains poorly understood. A possible role of the SNP rs8878 as a biomarker for CRC risk requires further investigations.

In conclusion, we demonstrated that the CXCL10 protein is upregulated in CRC tissue compared to paired normal tissue and that its plasma levels are higher in CRC patients compared to those in control subjects, which also reflects disseminated disease. Moreover, we observed an association between the rs8878 CXCL10 gene polymorphism and CRC risk in a Swedish population.

## Figures and Tables

**Table I:** Tissue and plasma levels of CXCL10 in colorectal cancer (CRC) patients and control subjects.

Variables	Cases	CXCL10 protein	P-value
Tissue levels (pg/mg)			<0.001
CRC tissue	99	482 (20–54,416)	
Paired normal tissue	99	171 (6–7,636)	
Plasma levels (pg/ml)			<0.01
CRC patients	104	734 (46–44,128)	
Control subjects	92	569 (9–2,492)	

Data are presented as median (range). Significance was set at P<0.05.

**Table II:** Genotypic and allelic distributions of rs8878 CXCL10 gene polymorphism in CRC patients and control subjects.

Genotype	CRC (n=366) n (%)	Controls (n=545) n (%)	Allele	CRC (n=732 alleles) n (%)	Controls (n=1,090 alleles) n (%)
T/T	28.7 (105)	24.0 (131)			
			T	52.6 (385)	47.6 (519)
T/C	47.8 (175)	47.1 (257)			
			C	47.4 (347)	52.4 (571)
C/C	23.5 (86)	28.9 (157)			

Colorectal cancer (CRC) patients vs. control subjects: genotypes overall and alleles, P=0.037.
